# Epidemiology and Determinants of Antirabies Vaccine Full-Dose Completion Among Patients Attending the Nyagatare District Hospital, Rwanda: A Facility-Based Cross-Sectional Study

**DOI:** 10.1155/jotm/2709448

**Published:** 2025-11-29

**Authors:** Jean Paul Hategekimana, Alexis Manishimwe, Gaetan Gatete, Emmanuel Nshimiyimana, Emmerance H. Gihozo, Allain P. Mutabazi, Patience Karemera, William Muturagara, Eddy K. Ndayambaje

**Affiliations:** ^1^Laboratory Department, Nyagatare Hospital, Nyagatare, Rwanda; ^2^Department of Epidemiology and Biostatistics, University of Rwanda, Kigali City, Rwanda; ^3^HIV/AIDs, Diseases Prevention and Control Department, Rwanda Biomedical Center, Kigali City, Rwanda; ^4^Department of Veterinary Medicine, University of Rwanda, Musanze, Rwanda; ^5^Clinical Services, Nyagatare Hospital, Nyagatare, Rwanda

**Keywords:** antirabies vaccine, dog bites, dropout, Nyagatare, postexposure prophylaxis

## Abstract

**Background and Aims:**

Antirabies vaccine postexposure prophylaxis (PEP) is effective in preventing rabies when administered promptly and in full. This study assessed factors associated with antirabies PEP full-dose completion among patients attending Nyagatare District Hospital.

**Methods:**

A facility-based cross-sectional study was conducted using data from patients who sought antirabies PEP at the hospital's emergency department in 2022. Patient files and registers were reviewed, and data were analyzed in STATA. Logistic regression was performed to identify factors associated with vaccine completion.

**Results:**

Of the 472 participants, 50.0% were under 16 years, 58.9% were male, and 63.6% lived in rural areas. Most (90.9%) had health insurance, 51.7% received PEP during the dry season, 11.0% received the antitetanus vaccine, and 82.2% had WHO wound Category II. Only 26.5% completed the full vaccination schedule, 16.1% missed appointments, and 57.4% dropped out before completion. Health insurance significantly increased the odds of completing PEP (AOR = 2.19, *p* = 0.032). Age, sex, and wound characteristics were not significantly associated with ARV full-dose completion.

**Conclusion:**

Compliance with the full antirabies vaccine course was low, with only a quarter of patients completing all five doses. Improving completion requires targeted interventions such as reminder systems, community education, provider counseling, and financial support.

## 1. Background

Rabies remains a public health burden and is a fatal viral infection where 100% of people who contract the disease die once clinical signs and symptoms appear, and this is most prevalent in tropical regions of the world [1].

Globally, an estimated 59,000 deaths annually from rabies are reported, 95% of which are from Africa and Asia. In these regions, about 80% of deaths are reported in poor rural communities, where many dogs remain unvaccinated, access to timely postexposure prophylaxis (PEP) is limited, and community awareness about rabies exposure is low [2].

Every year, more than 29 million people worldwide receive postexposure vaccinations that act to prevent hundreds of thousands of rabies deaths annually. Globally, the economic burden of dog-mediated rabies is estimated at USD 8.6 billion per year [3].

In 2015, the World Health Organization (WHO), the World Organization for Animal Health, the Food and Agriculture Organization of the United Nations, and the Global Alliance for Rabies Control targeted eliminating rabies-related human deaths by 2030 and ensuring a coordinated response to prevent rabies, integrated with the strengthening of human and veterinary health systems to reach the world's most underserved populations [4].

Prompt and complete antirabies PEP in the case of exposure to the virus strongly reduced rabies-related mortality and immunization failure [5]. PEP vaccines are given to patients based on the WHO wound classification Category II and Category III wounds, along with wound cleaning and additional rabies immunoglobulin (RIG) (Category III wounds only) [6].

Most of the patients who initiated antirabies vaccine (ARV) discontinued the vaccination on their own without completing the full course. No completion of PEP vaccine courses (doses) as scheduled was highlighted in many studies, which led to immunization failure and mortality [7, 8].

In 2016, Rwanda accounted for 413 dog bite cases, with an average of 54 dog bites per month recorded, and one death resulting from rabies was reported by the Rwanda Biomedical Center Epidemic Surveillance and Response Division [9].

ARV PEP is provided at the hospital based on the level of exposure to five courses of the ARV for a complete immunization that must be provided in 28 days; the first dose is given on the first day of a medical visit, the second dose on Day 3, the third dose on Day 7, the fourth dose on Day 14, and the fifth dose on Day 28 after the first dose, and it is administered intradermally and intramuscularly [10].

The vaccine shall be administered on the recommended appointment; a minor deviation is allowable, and a long deviation from the recommended schedule for several days may compromise the vaccine's effectiveness [11].

Although studies have examined aspects of rabies PEP in Rwanda [10] to the best of our knowledge, there has been a lack of research on full dose completion of PEP with the ARV among animal bite patients in the country. This study aimed to evaluate the factors associated with ARV PEP full dose completion among patients attending Nyagatare District Hospital, Rwanda 2022.

## 2. Methodology

### 2.1. Study Design and Period

This was an institutionally based cross-sectional study in which data from patients who sought ARV prophylaxis at the emergency department from 1^st^ January 2022 to 31^st^ December 2022 were used.

### 2.2. Study Setting

The study was conducted at Nyagatare Hospital, which is a secondary-level healthcare facility and is located in Nyagatare District in the Eastern Province of Rwanda. Nyagatare District has fertile soil, hence farming and cattle breeding are the main occupations of the population in this area. Due to its proximity to the Akagera National Park, half of this area falls under the Nyagatare District area and has brought humans and domestic and wild animals in proximity, hence increasing the incidence of wild animal bites in humans and domestic animals in the region [12]. Nyagatare Hospital is a public hospital that provides various packages of activities, including PEP, wound washing, and the provision of an ARV that is administered intramuscularly to rabies-exposed patients from every corner of the district premises and neighboring districts. The immunoglobulin is not provided. At the hospital, the vaccination for antirabies is not free; the patients are charged and have to pay out of pocket. Approximately two to four rabies-exposed patients are received at the emergency department daily. The patients' information is entered into the Open Medical Record System (OpenMRS), which is an open-source medical record system.

### 2.3. Study Participants

#### 2.3.1. Study Variables

##### 2.3.1.1. Sociodemographic Characteristics

Age (age groups), sex (male and female), availability of health insurance, urban or rural locality, and distance to the hospital.

##### 2.3.1.2. Clinical Characteristics

Wound categorization (WHO): Category II/III; number of wounds: single/multiple; wound management: antitetanus, antibiotic, and wound dressing (yes/no); and immunization status: number of doses received, completion (yes/no), timeliness (on-time/delayed/missed).

#### 2.3.2. Technical Definitions

• Compliant: rabies-exposed patients who completed five courses of immunization.
• Noncompliant: rabies-exposed patients who did not complete five courses of immunization.
• On the recommended appointment day: Participants who received the vaccine on the exact day it was scheduled.
• In allowable interval (< 7 days): Participants who received the vaccine within the recommended period, but slightly earlier or later (within 7 days).
• Before the recommended appointment: Participants who received the dose before the scheduled day.
• Late (more than 7 days): Participants who received the vaccine more than 7 days after the recommended date.
• Missed (drop-out): Participants who did not receive the dose at all.
• Respected rendezvous: respecting the immunization appointment date.
• Wound Category II*:* rabies-exposed patients who received only the ARV and/or no antibiotic.
• Wound Category III: rabies-exposed patients who received an ARV and wound dressing.
• Allowable days: 7 days from and appointment day

## 3. Data Sources/Measurement

### 3.1. Study Size

This study included all rabies-exposed patients who attended and sought the ARV PEP at the emergency department during the study period.

## 4. Data Collection

A nonrandom, purposive total population sampling approach was used. Data were extracted from the OpenMRS database and compiled in Microsoft Excel. All patients who received the ARV during the study period were identified from vaccination records. Patients with complete hospital records were included, while those with missing or inaccurate data, as well as those who sought care elsewhere, were excluded. Vaccination dates were cross-checked to ensure completeness, minimizing potential selection bias.

## 5. Data Analysis

Descriptive statistics were performed via proportions, means, and percentages to summarize the sociodemographic and clinical characteristics of the study participants. Bivariate analyses using the chi-square test were conducted to identify factors associated with full dose completion of the ARV. Variables with *p* < 0.2 in bivariate analyses were included in multivariate logistic regression to determine independent predictors of ARV full-dose completion. The outcome variable was ARV full-dose completion to the schedule, and independent variables included sociodemographic characteristics (age, sex, health insurance, locality, and distance to the hospital) and clinical characteristics (wound category, number of wounds, wound management, wound dressing, and immunization status). Excel was used for data collection and cleaning, and STATA Version 14.2 was used for analysis.

## 6. Ethical Approval

Ethical approval for this study was sought from the Nyagatare District Hospital Research and Ethics Committee. All extracted data were anonymized, and there were no individual identifiers. To ensure confidentiality, computer access was restricted by password protection. No informed consent nor assent was sought from the participants, as these data were secondary.

## 7. Results

A total of 472 participants attended the emergency department seeking the ARV as PEP; the median age was 16 years, and the interquartile range was 1–79 years.

### 7.1. Sociodemographic Characteristics of the Participants

Out of 472 participants, 236 (50.0%) were younger than 16 years. Males accounted for 278 (58.9%) of the participants, while females accounted for 194 (41.1%). The majority, 300 (63.6%), were from rural areas, and 172 (36.5%) were from urban areas. In terms of distance to the hospital, 241 (51.1%) lived less than 10 km away, whereas 230 (48.7%) traveled more than 10 km to reach Nyagatare Hospital. Regarding health insurance, 429 (90.9%) participants were treated with insurance, while 43 (9.1%) had no insurance. Half of the participants, 244 (51.7%), were bitten by animals during the dry season, and 228 (48.3%) during the rainy season.

### 7.2. Clinical Characteristics of the Participants

Out of 472 participants, only 52 (11.02%) were administered the antitetanus toxoid vaccine. Regarding antibiotic prescription, 94 (19.9%) received antibiotics such as cloxacillin 73.4%, amoxicillin and clavulanate 23.4%, metronidazole 2.13%, and doxycycline 1.06%. According to the WHO wound categorization, most of the participants, 388 (82.2%), presented with wound Category II and 84 (17.8%) wound Category III. According to the number of wounds, 398 (84.3%) had single wounds, 74 (15.7%) had multiple wounds, and 88 (18.6%) had their wounds dressed.

### 7.3. Description of Participants by the Completeness of the Immunization Course

Table 1 provides a detailed analysis of the completeness of a full five-dose antirabies vaccination among different demographic and clinical groups. The data revealed that, compared to older individuals, younger individuals, particularly those under 16 years, have the highest completion rate of 46.2%.

Out of the 278 male participants, 125 (45.0%) had completed the five doses, and 153 (55.0%) did not complete the five doses, while out of the 194 female participants, 76 (39.2%) completed the five doses, whereas 118 (60.8%) did not.

Based on participants' locality, out of the 172 urban participants, 68 (39.5%) completed the vaccination course, and 104 were counterparts; out of the 300 rural participants, 133 (44.3%) completed the vaccination course, and 167(55.7%) did not.

According to health insurance use, those with health insurance, that is, 188 (43.8%) completed the course and 241(56.9%) did not, whereas those with no health insurance, that is, 13 (30.2%) completed the vaccination course and 30 (69.8%) did not.

Proximity to healthcare facilities, such as being within 10 km of a district hospital, also contributes to a higher completion rate (43.2%). Seasonally, more people complete the vaccination during the rainy season (45.6%) than during the dry season (39.8%). Clinically, those who received the antitetanus vaccine (57.7%) or antibiotics (45.8%) were more likely to complete the antirabies vaccination. In addition, individuals with Category III wounds (53.6%) and those with multiple wounds (58.1%) had higher completion rates, possibly due to the perceived severity of their injuries. Wound dressing also plays a significant role, as 53.4% of individuals with dressed wounds completed the full vaccine schedule, indicating that healthcare interventions significantly increase the likelihood of completing the vaccination regimen.

### 7.4. ARV Dosing Interval Among Study Participants, Nyagatare Hospital, 2022

Table 2 shows that the adherence to the ARV dosing schedule among participants varied across the five doses in 2022, at Nyagatare Hospital. Among the participants, 472 participants received the first dose. For Dose 2, only 9 (1.9%) participants received the vaccine on the recommended day, while 355 (75.2%) received it within the allowable interval (less than 7 days), 33(7%) were late, and 15.9% missed the dose altogether. For Dose 3, 12 (4.3%) participants received the vaccine on the scheduled day, 69.6% within the allowable interval, 45 (11.3%) were late, and 59 (14.8%) missed the dose. By Dose 4, 15 (4.4%) received the vaccine on the recommended day, 218 (64.5%) within the allowable interval, while 42 (12.4%) were late, and 18% missed the dose. By Dose 5, adherence had further declined, with only 11 (4.0%) participants receiving the vaccine on the recommended day, 169 (61%) within the allowable interval, 9 (3.2%) late, and 76 (27.4%) missing the final dose. These data show a trend of decreasing adherence to the vaccine schedule, with a notable increase in missed doses as the series progressed (Table 2).

### 7.5. ARV Full-Dose Completion Status Among Study Participants, Nyagatare Hospital, 2022

**Figure 1:**
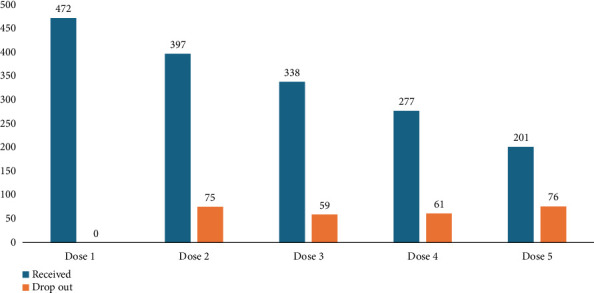
Antirabies vaccine full-dose completion status among study participants, Nyagatare Hospital, 2022.

Figure 1 illustrates dose completion among study participants for the second through fifth doses. A total of 472 participants received the first dose of the ARV. For the second dose, 397 participants (84.1%) were vaccinated, while 75 (15.9%) dropped out. By the third dose, 338 participants (71.6%) received the vaccine, and 59 (12.5%) dropped out. For the fourth dose, 277 participants (58.7%) were vaccinated, with 61 (12.9%) missing the dose. Finally, for the fifth dose, 201 participants (42.6%) received the vaccine, while 76 (16.1%) dropped out. This shows a progressive decline in dose completion, with the dropout rate increasing as the series progressed.

### 7.6. Factors Associated With Compliance With Antirabies PEP, Nyagatare Hospital, 2022

The analysis revealed that having health insurance significantly increased the odds of completing vaccination (AOR: 2.19, 95% CI: 1.07–4.49, *p* = 0.032), indicating that insurance is a strong predictor of vaccine completion Other predictors, such as wound dressing (AOR: 0.34, 95% CI: 0.03–3.71), had wide confidence intervals, suggesting potential limitations in precision. While other factors, such as age, sex, and wound characteristics, showed some trends, they were not statistically significant (Table 1).

## 8. Discussion

Half of the participants were less than 16 years old, which was similar to other findings from studies performed in Uganda and India, and this may be related to the playful activities and provocative behaviors of these teenagers such as beating, teasing, or handling encountered animal, which expose them to animal bites, especially dogs [13, 14] However, other studies have reported a higher risk of animal bites among older age groups, suggesting that age-related exposure patterns may vary across different settings [15, 16].

Our study revealed that males were the dominant patients of rabies exposure, which was similar to the findings from other studies [1, 14–19]. This may be related to the fact that males do more outdoor activities than females. Conversely, some studies have reported a higher proportion of female patients, possibly due to underreporting of dog bites among males [20].

Our study found that the majority of participants were from rural areas, and this can be attributed mainly to the large population of straying dogs and geographical status where most of the population's occupation consists of farming and cattle breeding in farms, and the proximity to Akagera National Park that increases the close contact of domestic and wild animals; these findings are similar to the findings of other studies performed elsewhere [15, 19, 21]; however, other studies have shown that most patients were from urban locality [22].

In the present study, approximately one-quarter of the participants completed the full vaccination by the recommended date, which was low compared to the findings of the study by Panda et al., which reported that compliance among rabies-exposed individuals was 6.7%, with a dropout rate of 45.5% [16]. Vaccination schedule dropout and delays have been reported in many studies, and these delays were attributed to forgetfulness and wound recovery [18].

Our study shows that the ARV full-dose completion declined steadily over time, with progressively more missed and delayed doses. This pattern, consistent with other studies [15, 18, 22, 23], reflects a combination of interrelated barriers. Financial constraints, particularly for uninsured patients facing high out-of-pocket costs, limit the ability to complete all five doses. Geographic and logistical challenges, such as travel distance and transport costs, reduce timely access to subsequent doses. Limited awareness of rabies risks and the benefits of completing the full vaccination course, coupled with cultural beliefs that dog bite wounds can be managed at home, further contribute to early discontinuation [19]. Health insurance mitigates some of these barriers by covering medical and transport expenses, explaining its strong association with full-dose completion in our study and others [22, 24].

## 9. Limitations

This study consisted of an analysis of data from hospital records. This lack of meaningful information, such as animal ownership, characteristics of biting animals, and rabies-exposed patients who did not visit the hospital, was excluded.

## 10. Conclusions

Our study found that compliance with the ARV was poor among rabies-exposed patients, where only a quarter of them remained compliant with all five dose courses. To improve ARV full-dose completion, targeted interventions are needed, including SMS or phone call reminders for upcoming doses, community outreach and education campaigns to raise awareness of rabies risks, training healthcare providers to counsel patients effectively, and subsidizing vaccination costs to reduce financial barriers.

## Figures and Tables

**Table 1:** Sociodemographic and clinical characteristics of the participants, Nyagatare Hospital, 2022.

Variable	Total *n* (%)	Complete *n* (%)	Incomplete *n* (%)	OR	95% CI	AOR	95% CI	*p* value
Sociodemographic characteristics								
Age in years								
** **< 16	236 (50.0)	109 (46.2)	127 (53.8)	Ref	—	—	—	—
** **16–30	120 (25.4)	46 (38.3)	74 (61.7)	0.72	0.46–1.13			
** **31–45	70 (14.8)	27 (38.6)	43 (61.4)	0.73	0.42–1.26			
** **46–60	30 (6.4)	13 (43.3)	17 (56.7)	0.89	0.41–1.92			
** **> 60	16 (3.4)	6 (37.5)	10 (62.5)	0.70	0.25–1.99			
Sex								
** **Female	194 (41.1)	76 (39.2)	118 (60.8)	Ref	—	—	—	—
** **Male	278 (58.9)	125 (45.0)	153 (55.0)	1.27	0.87–1.84	1.24	0.85–1.82	0.265
Locality								
** **Rural	300 (63.6)	133 (44.3)	167 (55.7)	Ref	—	—	—	—
** **Urban	172 (36.5)	68 (39.5)	104 (60.3)	0.82	0.56–1.20			
Distance to DH								
** **< 10 km	241 (51.1)	104 (43.2)	137 (56.9)	Ref	—	—	—	—
** **≥ 10 km	230 (48.7)	97 (42.2)	133 (57.8)	0.96	0.67–1.38	0.94	0.64–1.37	0.734
Available health insurance								
** **No	43 (9.1)	13 (30.2)	30 (69.8)	Ref	—	—	—	—
** **Yes	429 (90.9)	188 (43.8)	241 (56.2)	1.8	0.91–3.54	**2.19**	**1.07–4.49**	**0.032**
Seasonality								
** **Dry	244 (51.7)	97 (39.8)	147 (60.3)	Ref				
** **Rainy	228 (48.3)	104 (45.6)	124 (54.4)	1.27	0.88–1.83	1.22	0.83–1.78	0.312
Clinical characteristics of participants								
Antitetanus toxoid vaccine								
** **No	420 (89.0)	171 (40.7)	249 (59.3)	Ref	—	—	—	—
** **Yes	52 (11.0)	30 (57.7)	22 (42.3)	1.99	1.10–3.56	1.72	0.92–3.23	0.092
Antibiotic								
** **No	378 (80.1)	158 (41.8)	220 (58.2)	Ref	—	—	—	—
** **Yes	94 (19.9)	43 (45.8)	51 (54.3)	1.17	0.74–1.85	0.96	0.57–1.61	0.865
WHO wound category								
** **Cat II	388 (82.2)	156 (40.2)	232 (59.8)	Ref	—	—	—	—
** **Cat III	84 (17.8)	45 (53.6)	39 (46.4)	1.72	1.06–2.76	1.85	0.24–14.45	0.558
Wound dressed								
** **No	384 (81.4)	154 (40.1)	230 (59.9)	Ref	—	—	—	—
** **Yes	88 (18.6)	47 (53.4)	41 (46.6)	1.71	1.07–2.72	0.34	0.03–3.71	0.374
Number of wounds								
** **Multiple	74 (15.7)	43 (58.1)	31 (41.9)	Ref	—	—	—	—
** **Single	398 (84.3)	158 (39.7)	240 (60.3)	0.47	0.28–0.79	0.28	0.08–1.03	0.055

*Note:* The bold values are the statistically significant values.

**Table 2:** Antirabies vaccine dosing interval among study participants, Nyagatare Hospital, 2022.

Dose received	Dose 1	Dose 2 *n* (%)	Dose 3 *n* (%)	Dose 4 *n* (%)	Dose 5 *n* (%)
On the recommended appointment day	472	9 (1.9)	17 (4.3)	15 (4.4)	11 (4.0)
In allowable interval days (< 7 Days)	0	355 (75.2)	277 (69.6)	218 (64.5)	169 (61.0)
Before the recommended appointment	0	0 (0)	0 (0)	2 (0.6)	12 (4.3)
Late (more than 7 days)	0	33 (7.0)	45 (11.3)	42 (12.4)	9 (3.2)
Missed (drop-out)	0	75 (15.9)	59 (14.8)	61 (18.0)	76 (27.4)

## Data Availability

The data that support the findings of this study are available on request from the corresponding author. The data are not publicly available due to privacy or ethical restrictions.

## Nomenclature

OR: Odds ratio
PEP: Postexposure prophylaxis
ARV: Antirabies vaccine
WHO: World Health Organization
